# β-cell-specific overexpression of adiponectin receptor 1 does not improve diabetes mellitus in Akita mice

**DOI:** 10.1371/journal.pone.0190863

**Published:** 2018-01-05

**Authors:** Jungmi Choi, Hatasu Kobayashi, Hiroko Okuda, Kouji H. Harada, Midori Takeda, Hiroyuki Fujimoto, Shunsuke Yamane, Daisuke Tanaka, Shohab Youssefian, Nobuya Inagaki, Akio Koizumi

**Affiliations:** 1 Department of Health and Environmental Science, Graduate School of Medicine, Kyoto University, Kyoto, Japan; 2 Department of Biomedical Sciences, College of Life and Health Sciences, Chubu University, Kasugai, Japan; 3 Radioisotope Research Center, Kyoto University, Kyoto, Japan; 4 Department of Diabetes, Endocrinology and Nutrition, Graduate School of Medicine, Kyoto University, Kyoto, Japan; 5 Laboratory of Molecular Biosciences, Graduate School of Medicine, Kyoto University, Kyoto, Japan; Virgen Macarena University Hospital, School of Medicine, University of Seville, SPAIN

## Abstract

Adiponectin, a metabolically-active cytokine secreted from adipose tissue, is reported to have anti-apoptotic effects on β-cells as well as anti-hyperglycemic effects through adiponectin receptor signaling. However, the anti-apoptotic effects of adiponectin on β-cells have not been confirmed in established diabetic models, and the anti-hyperglycemic effects and their associated signal cascades remain controversial. To investigate the effects of adiponectin on β-cell protection and its down-stream signaling events, we have generated β-cell-specific rat insulin promoter (RIP)-AdipoR1 transgenic mice (AdipoR1 mice), in which the adiponectin receptor, AdipoR1, is overexpressed in β-cells in a manner synchronous with insulin demand. AdipoR1 mice were then mated with Akita mice, a diabetes model in which β-cell apoptosis results from endoplasmic reticulum (ER) stress. AdipoR1 protein expression and localization in islets from AdipoR1 mice as well as in an AdipoR1-transfected mouse insulinoma cell line were confirmed, as was the activation of both AMPK and Akt in AdipoR1 mice by adiponectin. Nevertheless, there were no significant differences in Ad lib feed and fasting blood glucose levels, or in glucose tolerance tests, between Akita mice [Ins2Akita (C96Y) +/- mouse model] and AdipoR1/Akita and from 4 weeks to 10 weeks of age. Similarly, pancreatic insulin contents of AdipoR1/Akita mice were not significantly different from those in Akita mice from 15 to 20 weeks of age, but they were significantly lower than in wild-type mice. Immunostaining for insulin and subsequent electron microscopy showed that β-cell destruction in AdipoR1/Akita mice was not markedly improved in comparison with that in Akita mice. Serum adiponectin concentrations were confirmed to be extremely high (> 30 μg / ml) compared with the Kd value (0.06 μg / ml) in all mouse groups at 15 to 20 weeks of age. Therefore, although the physiological levels of adiponectin are sufficient to activate AMPK and Akt when AdipoR1 is overexpressed in β-cells, yet adiponectin cannot protect β-cells in Akita mice from ER stress-induced destruction.

## Introduction

Diabetes mellitus is characterized by chronic hyperglycemia due to insulin deficiency and insulin resistance. Type 1 diabetes is characterized by pancreatic β-cell destruction, and type 2 diabetes by impaired insulin secretion and insulin resistance. Recent studies have demonstrated that even in type 2 diabetes, pancreatic β-cells are destroyed by apoptosis, suggesting that a decrease in β-cell mass is common to both types of diabetes mellitus [1]. Thus, the recovery of β-cell mass may be a future strategy in the prevention and treatment of type 1 and type 2 diabetes mellitus [2, 3].

Adiponectin, a 30 kDa protein secreted predominantly by adipose tissue, is known to activate fatty acid oxidation and glucose metabolism [4, 5]. Adiponectin has also been reported to have anti-apoptotic effects on pancreatic β-cells, and this has been demonstrated using MIN6 cells, a β-cell line, as well as in isolated mouse islets, and in a mouse model of inducible apoptosis of pancreatic β-cells by conditional activation of caspase 8 [6–8]. Recently, it was also reported that adiponectin receptor signaling increases ceramidase activity and thereby elevates the levels of the anti-apoptotic metabolite, sphingosine-1-phosphate [6]. Although it has been proposed from such studies that adiponectin has anti-apoptotic effects on β-cells, yet there has been no confirmation of this in established diabetic models.

Furthermore, adiponectin has been reported to have anti-hyperglycemic effects, mediated through specific signaling pathways, in diabetic mouse models. However, both these findings are controversial, with some studies showing that adiponectin can lower blood glucose in diabetic mice [9–11], whereas others have shown that it does not [12, 13]. In addition, although these anti-hyperglycemic effects have been attributed to the activation of AMP-activated protein kinase (AMPK) and Akt, some reports demonstrate AMPK activation [6,14], while other do not [8].

Adiponectin receptors, especially adiponectin receptor 1 (AdipoR1), are expressed in pancreatic islets [15]. From the viewpoint of reaction kinetics, changes of adiponectin level may not have any influence *in vivo*, since the adiponectin receptor dissociation constant (Kd) is known to be extremely low (0.06 μg/ ml) in comparison with the physiological adiponectin concentration (6 μg/ml) [14]. The dissociation of adiponectin is represented in Eq1 [16]:

$$V=\frac{\left[A][R\right]}{Kd+[A]}$$ (1)

where [A] is adiponectin concentration, [R] is the adiponectin receptor concentration, V is the effect of adiponectin, and Kd is adiponectin receptor dissociation constant. Since Kd is much lower than [A] (Eq 2), Eq 1 can be approximated as Eq 3, thereby deriving Eq 4.

Kd≪[A] (2)

$$V≅\frac{\left[A][R\right]}{\left[A\right]}$$ (3)

V≅[R] (4)

This suggests that the effect of adiponectin *in vivo* is more greatly affected by the adiponectin receptor levels than the adiponectin concentration, and that the level of adiponectin would have no effect even at levels that fluctuate within the μg/ml range. In contrast, the effects of adiponectin would be expected to be easily perturbed with changes in adiponectin receptor levels.

Our main objective in this study was to investigate whether adiponectin can protect pancreatic β-cells from destruction in a diabetic mouse model. In advanced diabetes, unavoidable β-cell mass death has promoted research towards the protection of β-cell mass, yet such an approach has been hampered by the absence of appropriate models. We, therefore, addressed our objective by the development of β-cell-specific AdipoR1 transgenic (Tg) mice (AdipoR1 mice), and thereafter AdipoR1/Akita mice [Ins2Akita (C96Y) +/- mouse model] in which β-cell destruction results from endoplasmic reticulum (ER) stress. We assumed that these AdipoR1 Tg mice would enable the effects of adiponectin to be addressed under conditions of AdipoR1 overexpression, thereby eliminating any uncertainties adherent to uncontrolled AdipoR1 levels. Our results clearly demonstrate that despite the correct expression and localization of AdipoR1 in β-cells of Tg mice, as well as the expected activation of the AMPK and Akt signaling pathways by adiponectin in islets of Tg mice, adiponectin was unable to protect the β-cells from ER stress-induced destruction.

## Materials and methods

### β-cell-specific AdipoR1 transgene vector construction

The transgene vector construct consisted of the rat insulin promoter (RIP) placed upstream of the mouse *AdipoR1* coding sequence fused in-frame to the enhanced green fluorescent protein (EGFP) and terminated by the SV40 poly(A) sequence. Here, the *AdipoR1* coding sequence was first amplified by RT-PCR from C57BL/6 mouse liver RNA using the SuperScript III One-Step RT-PCR System with Platinum Taq DNA Polymerase (Invitrogen, Carlsbad, CA) and cloned into the pCR4Blunt-TOPO vector. The *AdipoR1* and *EGFP* fragments were independently PCR amplified with Q5 DNA polymerase (New England BioLabs, Ipswich, MA) using appropriately-designed In-Fusion primers that would place the amplified *Sal*I-AdipoRI fragment in-frame and upstream of the EGFP-*Not*1 fragment. The two amplified fragments were then co-integrated into the *Sal*I-*Not*I double-digested RIP-Timer1 vector (Addgene [https://www.addgene.org], DM#285) using the In-Fusion HD Cloning Kit (Clontech, Mountain View, CA). Constructs were confirmed by DNA sequence analysis. The resulting transgenic mice carrying RIP-*AdipoR1* were expected to overexpress AdipoR1 in β-cells, and its level to be regulated in a manner synchronous with insulin demand [17].

### Cell cultures and protein extraction/tissue fractionation

Mouse insulinoma 6 (MIN6 cells) were obtained from Dr. Junichi Miyazaki (Osaka University Medical School), and used between passages 10 and 18 in all our experiments. Cells were cultured in high glucose (25 mM) Dulbecco's modified Eagle's medium (Invitrogen) supplemented with 10% fetal calf serum (Sigma, St. Louis, MO) and 1% penicillin-streptomycin (Invitrogen) under 5% CO_2_ and 95% air at 37°C. The MIN6 cells were transiently transfected with a total of 0.75 μg of control (empty RIP-timer 1 vector), AdipoR1 or EGFP-tagged AdipoR1 (AdipoR1-EGFP) in antibiotic-free medium and seeded on 24-well plates using 2.5 μl of Lipofectamine 2000 reagent (Invitrogen). After 48 h of transfection, AdipoR1 was stimulated in the MIN6 cells by treatment with 5 μg/ml full length Adiponectin (fAd) (R&D Systems Inc., Minneapolis, MN) for 10 min under serum-free conditions as described previously [8]. The cells were washed twice with phosphate-buffered saline (PBS), and then lysed for 30 min at 4°C with lysis buffer (140 mM NaCl, 10 mM Tris-HCl (pH 7.4), 10% glycerol, 1% Nonidet P-40, protease inhibitor (Nacalai, Kyoto, Japan) and PhosSTOP phosphatase inhibitor (Roche Diagnostic GmbH, Mannheim, Germany). The cells were scraped from the dishes, collected by centrifugation at 15,000 x *g* for 15 min at 4°C, and the cytosolic and membrane fractions prepared using the Qproteome Cell Compartment Kit (Qiagen, Valencia, CA, USA).

### β-cell-specific AdipoR1 Tg mice production and experimental animals

The transgene construct was digested with *Ssp*I, located in the vector region, and the linearized 5.5 kb DNA fragment purified and then microinjected into fertilized C57BL/6 mouse eggs to generate Tg mice. Genotypes of the Tg offspring were determined by PCR using the primers (Forward, 5’-GCA CCT CTT ATG GAG AGT TGC TG-3’; Reverse, 5’-CAA ACC ACA ACT AGA ATG CAG TG-3’). Akita and WT mice were purchased from Japan SLC (Hamamatsu, Japan). AdipoR1 mice (β-cell-specific *AdipoR1* Tg) were bred with Akita mice (*Ins2*^+/C96Y^) to obtain AdipoR1/Akita mice (β-cell-specific *AdipoR1* Tg, *Ins2*^+/C96Y^). Experiments in the present study were therefore performed on four groups of male mice in a C57BL/6 background: (1) AdipoR1/Akita mice; (2) AdipoR1 mice; (3) Akita mice; (4) WT mice. Mice were maintained on a standard chow diet with free access to food and water, and sacrificed by CO_2_ asphyxiation. Care of animals and all experimental procedures were in accordance with the Animal Welfare Guidelines of Kyoto University. The experimental protocol was authorized by the Internal Animal Welfare Committee at Kyoto University (approval no., Med Kyo16595; approval date, 2016/3/31).

### Islet isolation and protein extraction/tissue fractionation

Under anesthesia, 2 ml of Hanks balanced salt solution (HBSS) containing 1 mg collagenase was injected into the common bile duct of the donor mice (WT, AdipoR1 mice). The distended pancreas was isolated and digested for 30 min at 37°C. Islets, from a total of 6 mice at 10–20 weeks of age, were purified by Histopaque-density centrifugation, and transferred into RPMI 1640 medium containing 11.1mM glucose. After 45 min of incubation, islets were stimulated by fAD for 10 min, washed twice with PBS, and total lysates prepared using Cellytic MT (Sigma) supplemented with protease inhibitor (Nacalai) and PhosSTOP phosphatase inhibitor (Roche Diagnostic GmbH).

### Immunoblotting

Protein concentrations were determined according to the Bradford method using BSA as standard. Cell lysates (10–20 μg per sample) were then separated by 5–20% SDS-PAGE with known molecular weight markers (Bio-Rad, California, USA) and transferred onto polyvinylidene fluoride membranes by standard procedures. Membranes were incubated in blocking solution (5% skim milk or BSA in TBS-T) for 3 h at room temperature, and hybridized overnight at 4°C with primary antibodies against vinculin (Sigma) (1:4000), AdipoR1 (Immuno-Biological Laboratories Inc., Minneapolis MN) (2 μg /ml), and also Akt (1:1000), phospho-Akt (s473 or t308) (1:1000), AMPK (1:1000), phospho-AMPK (1:1000) all from Cell Signaling Technology (CST, Danvers, MA, USA). The membranes were washed three times with TBS-T and then incubated with either anti-rabbit (Sigma) (1:10000) or anti-mouse (Abcam, Cambridge, U.K.) (1:10000) secondary antibodies for 1 h at room temperature. After washing, immunodetection was performed using an enhanced chemiluminescence solution (Clarity Max Western ECL Substrates, Bio-Rad) and scanned on Fujifilm LAS-3000 system and quantified using Image-J software (National Institutes of Health).

### Reverse-transcription PCR

Total RNA was isolated from mouse pancreas with the RNeasy Mini kit (Qiagen) with RNase-free DNase digestion according to the manufacturer’s instructions. Reverse-transcription (RT) reactions were conducted with 2 μg of total RNA using the High Capacity cDNA Reverse Transcription Kit (Thermo Fisher Scientific, Waltham, MA) according to the manufacturer's instructions. Primers for amplification of the expressed AdipoR1-EGFP transgene were those used for In-Fusion cloning: Forward, 5’-TAA CTC TAG AGT CGA CCG CCA CCA TGT CTT TCC CA-3’; Reverse, 5’-GCC CTT GCT CAC CAT GAG AAG GGA GTC GTC GGT-3’ (expected size; 1910 bp). β-actin was used as an internal amplification control.

PCR amplifications were performed as follows: 5 min at 95°C followed by 40 cycles consisting of 45 sec at 95°C, annealing 58°C for 45 sec, 2 min at 74°C, and an additional elongation step for 7 min at 72°C using KOD DNA polymerase (TOYOBO, Osaka, Japan). The PCR-amplified products were separated on a 1.0% agarose gel and visualized by ethidium bromide staining.

### Measurement of blood glucose

Ad libitum feed blood glucose and 16 h fasting blood glucose were measured using the Glutest Neo Super blood glucose meter (Sanwa chemical, Nagoya, Japan) once a week by collecting blood samples from the mouse tail. Glucose tolerance testing (GTT) was performed on 6- and 10-week-old mice after 16 h fasting, followed by an intraperitoneal injection of 1.5 g/kg glucose. All GTT test results higher than 33.3 mM were recorded as 33.3 mM.

### Measurement of pancreatic insulin contents

Mice were sacrificed at 15–20 weeks of age. Collected pancreatic samples were homogenized in acid ethanol (75% ethanol, 1.5% HCl) and extracted at 4°C overnight. The extracts were centrifuged, and insulin concentration of the supernatants determined using an enzyme-linked immunosorbent assay (ELISA) kit (Shibayagi, Gunma, Japan).

### Pathological investigations

Mice were sacrificed at 15–20 weeks of age. The pancreas was excised and processed for immunostaining, RT PCR and electron microscopy. To confirm pancreatic β-cell-specific overexpression of EGFP-tagged AdipoR1, fresh frozen sections were prepared using OCT compound (Tissue-Tek, Sakura Finetek, Torrance, CA) and double-immunostained with rabbit anti-GFP antibody (Abcam) and guinea pig anti-insulin antibody (DAKO, Carpinteria, CA). The sections were incubated with Alexa 594-labeled anti-rabbit IgG and Alexa 488-labeled anti-guinea pig IgG antibody (Molecular Probes, Eugene, OR) and observed by confocal laser microscopy (TCS SP8, Leica, Wetzlar, Germany). For insulin immunostaining to estimate β-cell mass, the pancreas was fixed in 10% formaldehyde, embedded in paraffin, and sectioned. The sections were immunostained with guinea pig anti-insulin antibody (DAKO), followed by incubation with biotinylated anti-guinea pig IgG (Vector Labs, Burlingame, CA). Detection was performed by ABC/DAB immunohistochemistry using the ABC kit (Vectastain, Vector Labs). For quantification, the islet area and insulin positive cells were measured on more than 20 islets per pancreas in five mice per genotype using Image-J software (National Institutes of Health.).

For electron microscopy, pancreas was fixed in 2% glutaraldehyde and post-fixed in 1% osmium tetroxide. The samples were dehydrated through a graded ethanol series, embedded in Epon, sectioned with an ultramicrotome (EM UC6, Leica), and stained with lead citrate. Sections were observed with an H-7650 transmission electron microscope (Hitachi, Tokyo, Japan).

### Serum adiponectin levels

Blood samples were obtained from mice, and serum was separated by centrifugation. Serum adiponectin levels were evaluated using a mouse adiponectin ELISA kit (Bio Vendor, Brno, Czech Republic).

### Statistical analysis

Results are presented as the mean ± standard error of the mean (SEM). Differences between the means of four groups of animals (AdipoR1/Akita, Akita, AdipoR1 and WT) or three groups of MIN6 cells (Control, AdipoR1 and Adipor1-EGFP) were evaluated using One-way ANOVA. When One-way ANOVA was significant, i.e., p<0.05, then Tukey’s honestly significant difference test was conducted to detect different groups. All statistical analyses were conducted using STATISTICA software (StatSoft). *P* < 0.05 was considered statistically significant.

## Results

### AdipoR1-EGFP overexpression and signaling functions in MIN6 cells

**Fig 1:**
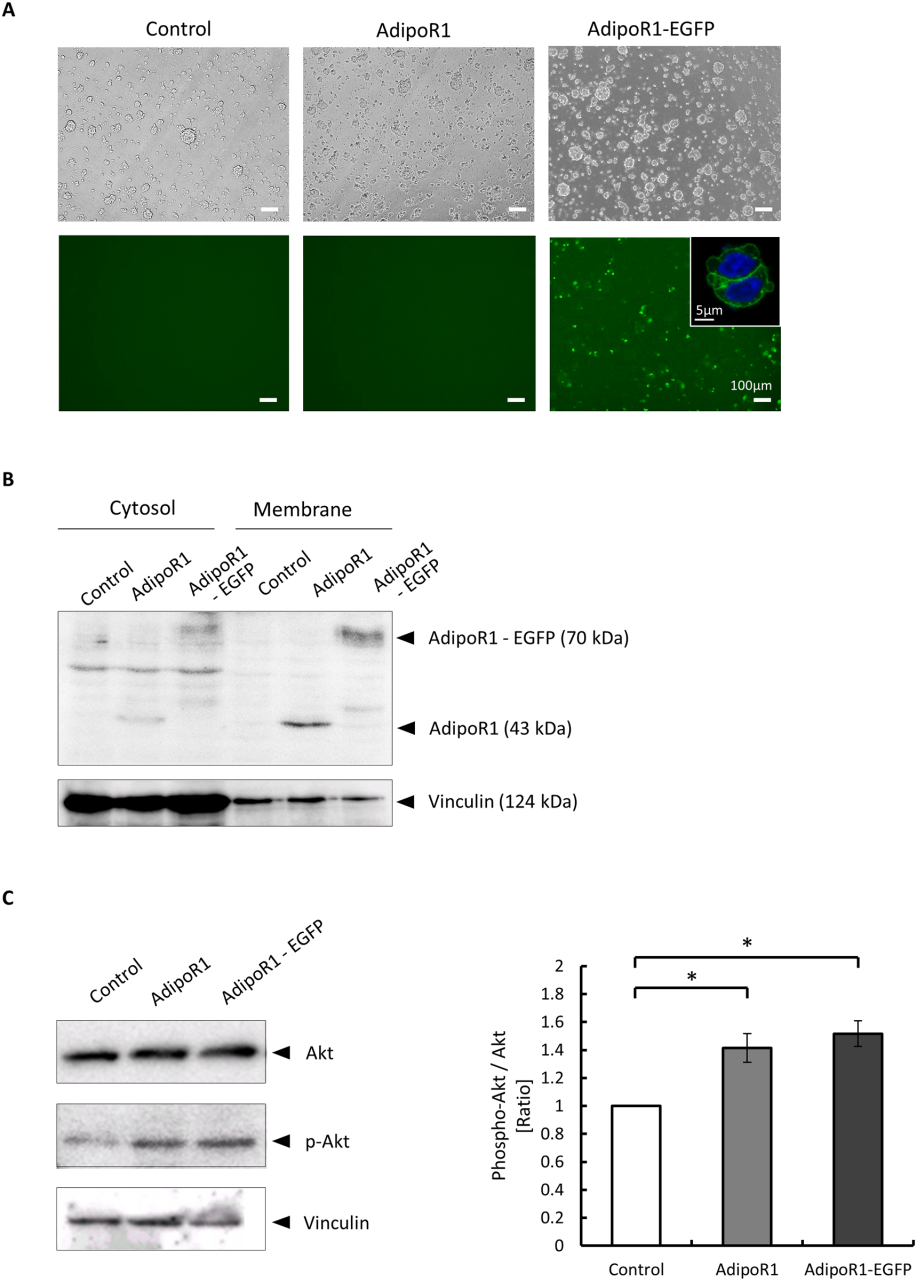
EGFP-tagged AdipoR1 protein expression and signaling function in MIN6 cells. (A) Representative fluorescence images of MIN6 cells transfected with empty vector (control), AdipoR1 or EGFP-tagged AdipoR1 (AdipoR1-EGFP) vectors. The scale bars represent the indicated dimensions. (B) Western blot analysis of cytosolic and membrane fractions of MIN6 cells transfected with control, AdipoR1or AdipoR1-EGFP vectors using anti-AdipoR1 antibody. Expected protein sizes are AdipoR1 (43 kDa) and AdipoR1-EGFP (70 kDa). (C) Akt phosphorylation in MIN6 cells transfected with control, AdipoR1 or AdipoR1-EGFP vectors. MIN6 cells were stimulated with 5μg/ml fAd in serum-free medium after transfections. Representative Western blots for Akt (60 kDa), phospho-Akt (Ser^473^) (60 kDa) and vinculin (124 kDa), as a loading control, are shown (left panel). Quantified values represent the ratios (compared with control) of phospho-Akt/Akt (right panel). Data are represented as mean ± SEM of four independent experiments. *p < 0.05, by ANOVA followed by Tukey’s honestly significant difference test compared with control.

We first confirmed the overexpression of EGFP-tagged AdipoR1 (AdipoR1-EGFP) by fluorescence microscopy using MIN6 cells transfected with control, AdipoR1 or AdipoR1-EGFP vectors. As shown in Fig 1A, AdipoR1-EGFP-transfected cells exhibited a strong green fluorescence, particularly along the plasma membrane, whereas control and AdipoR1-transfected cells displayed no such fluorescence, suggesting correct expression of the AdipoR1-EGFP fusion protein. Western blot analysis using anti-AdipoR1 antibody confirmed AdipoR1 (43 kDa) and AdipoR1-EGFP (70 kDa) overexpression in the AdipoR1- and AdipoR1-EGFP-transfected MIN6 cells, respectively (Fig 1B). The overexpressed AdipoR1 and AdipoR1-EGFP proteins were localized to both the cytosolic and membrane fractions, but predominantly to the membrane fractions (Fig 1B).

To determine whether the C-terminal EGFP tag had any influence on AdipoR1 signaling, we examined the extent of Akt phosphorylation, which reportedly functions downstream of adiponectin signaling in pancreatic β-cells [8], in the MIN6 cells overexpressing AdipoR1-EGFP or AdipoR1 after stimulation with full length adiponectin (fAD). We confirmed that not only AdipoR1 but also AdipoR1-EGFP significantly increased Akt phosphorylation in MIN6 cells in comparison with the control vector (Fig 1C). The comparable levels of Akt phosphorylation in the AdipoR1 and AdipoR1-EGFP overexpressing cells (Fig 1C), confirm that the EGFP tag is unlikely to have affected the downstream signaling pathways mediated by AdipoR1.

### AdipoR1-EGFP overexpression and signaling functions in Tg mice islets

**Fig 2:**
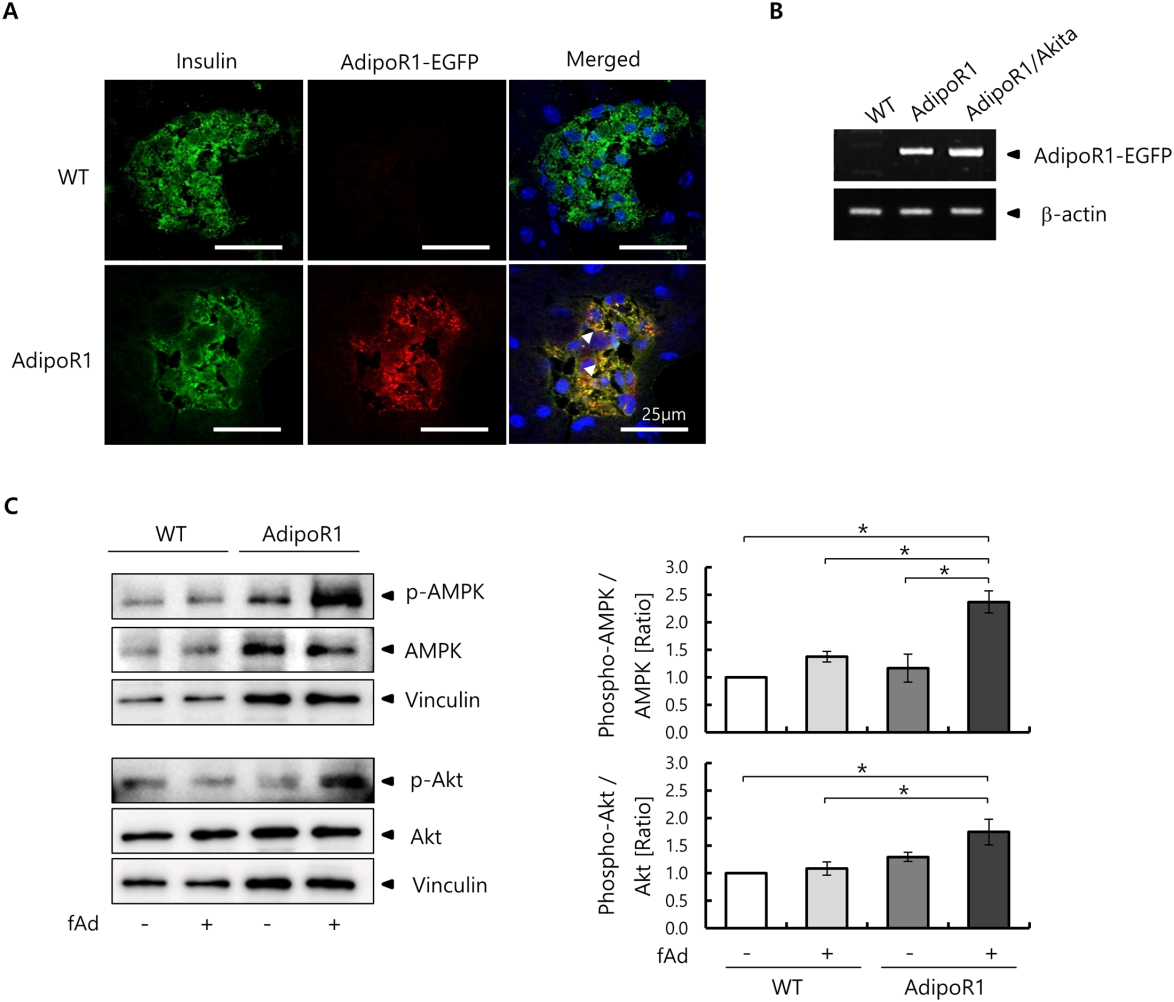
β-cell specific AdipoR1-EGFP overexpression and signaling functions in AdipoR1 mice. (A) Pancreatic tissue samples were obtained from AdipoR1 and WT mice at 8 weeks of age. Pancreatic tissue sections were double immunostained for EGFP and insulin. DNA was stained by DAPI. Representative single-channel fluorescence and merged images are shown. Red, green and blue staining correspond to signals for AdipoR1-EGFP, insulin and DNA, respectively. Similar results were obtained from three independent experiments. The scale bars indicate 25 μm. (B) RT-PCR analysis of AdipoR1-EGFP transcripts (1910 bp) in pancreata of WT, AdipoR1 and AdipoR1/Akita mice. RT-PCR products were subjected to 1% agarose gel electrophoresis. β-actin (474 bp) was used as a loading control. (C) AMPK and Akt phosphorylation in WT and AdipoR1 mice islets stimulated with or without 5 μg/ml fAd. Representative Western blots for phospho-AMPK and AMPK (62 kDa), phospho-Akt and Akt (60 kDa), and vinculin (124 kDa) as a loading control, are shown (left panel). Quantified values represent the ratios (compared with control untreated WT mice) of phospho-AMPK/AMPK and phospho-Akt/Akt (right panel). Data are presented as mean ± SEM of three independent experiments. *p < 0.05, by ANOVA followed by Tukey’s honestly significant difference test compared with control mice.

We examined the overexpression of AdipoR1-EGFP in β-cells of Tg mice by double-immunostaining for EGFP and insulin. AdipoR1 mice, but not WT mice, showed co-expression of AdipoR1-EGFP and insulin in pancreatic islets (Fig 2A). We also determined the transcript levels of the AdipoR1-EGFP transgene in the pancreas of WT, AdipoR1 and AdipoR1/Akita mice. As expected, RT-PCR products of AdipoR1-EGFP mRNA were detected in the pancreas of AdipoR1 and AdipoR1/Akita mice, but not of WT mouse (Fig 2B). These results confirm the pancreatic β-cell-specific overexpression of AdipoR1-EGFP in AdipoR1 and AdipoR1/Akita mice.

We then tested the downstream signaling functions in islets derived from Tg mice in response to fAd, and confirmed that fAd activated both AMPK and Akt in islets from the EGFP-tagged AdipoR1 Tg mice. AMPK phosphorylation was significantly increased in AdipoR1 mouse islets stimulated with fAd in comparison with unstimulated islets from AdipoR1 mice as well as islets from WT mice (Fig 2C). However, there were no significant differences in AMPK phosphorylation levels between WT mouse islets stimulated with fAd and unstimulated islets from WT mice. Furthermore, Akt phosphorylation in AdipoR1 mice islets stimulated by fAd was not significantly higher than unstimulated islets from AdipoR1 mice, but were significantly higher than levels in WT mice islets (Fig 2C).

Overall, these results clearly demonstrate that overexpression of AdipoR1 in pancreatic β-cell is associated with increased AMPK and Akt phosphorylation by adiponectin, and that physiological levels of adiponectin are insufficient to activate these signal molecules unless AdipoR1 is overexpressed.

### Body weight over time

**Fig 3:**
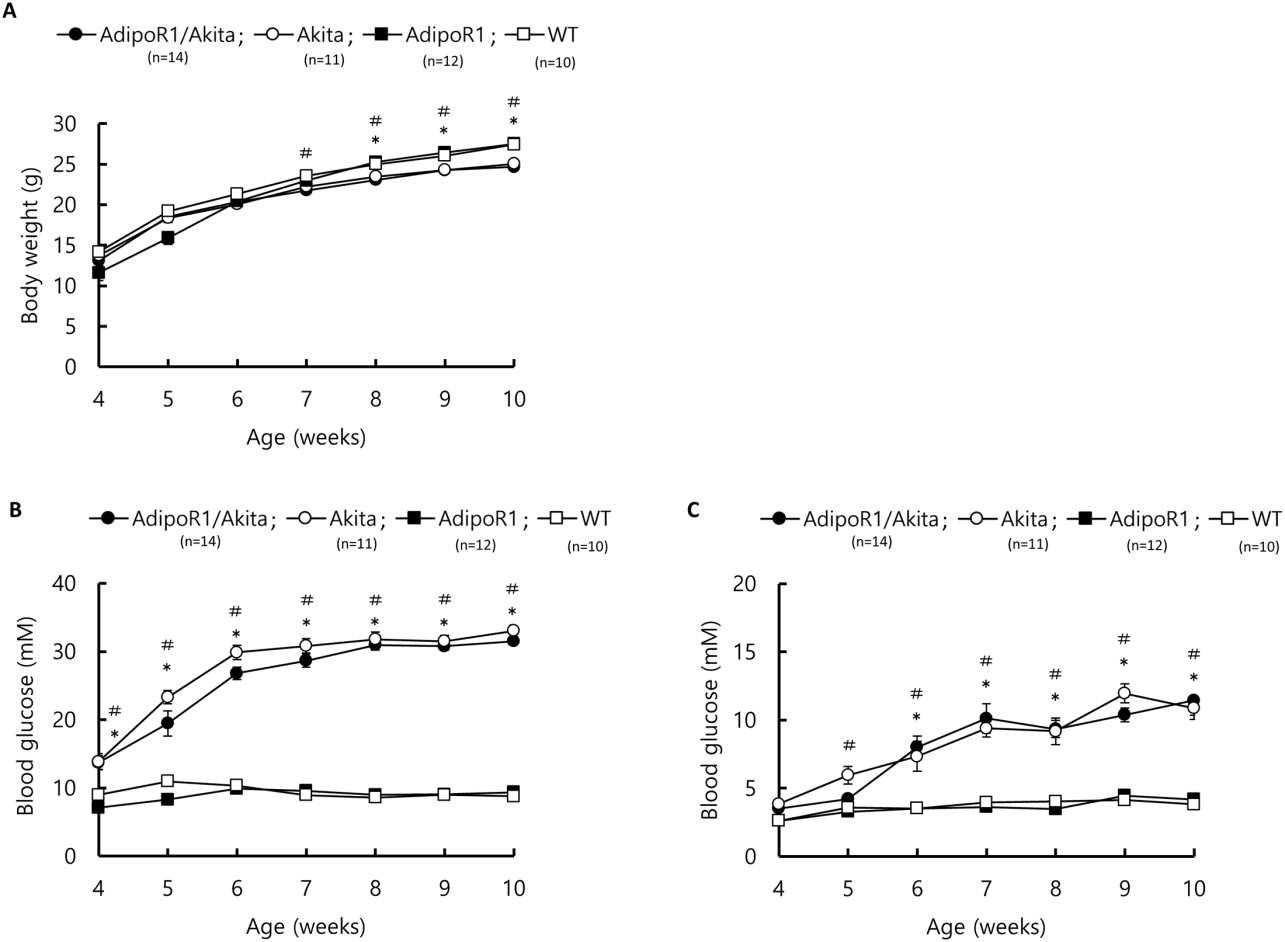
Growth curves and blood glucose of AdipoR1/Akita, AdipoR1, Akita and WT mice. Growth curves and blood glucose levels of AdipoR1/Akita (n = 14), Akita (n = 11), AdipoR1 (n = 12), and WT (n = 10) mice. (A) Time course of body weight changes of mice between 4 and 10 weeks of age. Blood glucose after (B) an Ad lib feed and (C) a 16 h fast in mice between 4 and 10 weeks of age. Values are expressed as mean ± SEM. There were no significant differences between AdipoR1/Akita and Akita mice, nor between AdipoR1 and WT mice, whereas there were significant differences between AdipoR1/Akita vs AdipoR1 (* p<0.05), and Akita and WT (# p<0.05) mice by ANOVA followed by Tukey’s honestly significant difference test.

All four groups of experimental mice exhibited an increase in body weight between 4 and 10 weeks of age (Fig 3A). During this period, there were no significant differences in body weight between the AdipoR1/Akita and Akita mice, nor between AdipoR1 and WT mice. However, the body weights of both AdipoR1/Akita and Akita mice were significantly lower than AdipoR1 and WT mice.

### Blood glucose levels and glucose tolerance

From 4 to 10 weeks of age, there were no significant differences in either Ad lib feed blood glucose levels or 16 h fast blood glucose levels between AdipoR1/Akita and Akita mice, nor between AdipoR1 and WT mice (Fig 3B and 3C). However, the Ad lib feed and 16 h fast blood glucose levels of AdipoR1/Akita mice were significantly higher than that of AdipoR1 mice after 4 weeks of age and 6 weeks of age, respectively. Similarly, Akita mice showed significantly higher Ad lib feed and 16 h fast blood glucose levels than WT mice.

**Fig 4:**
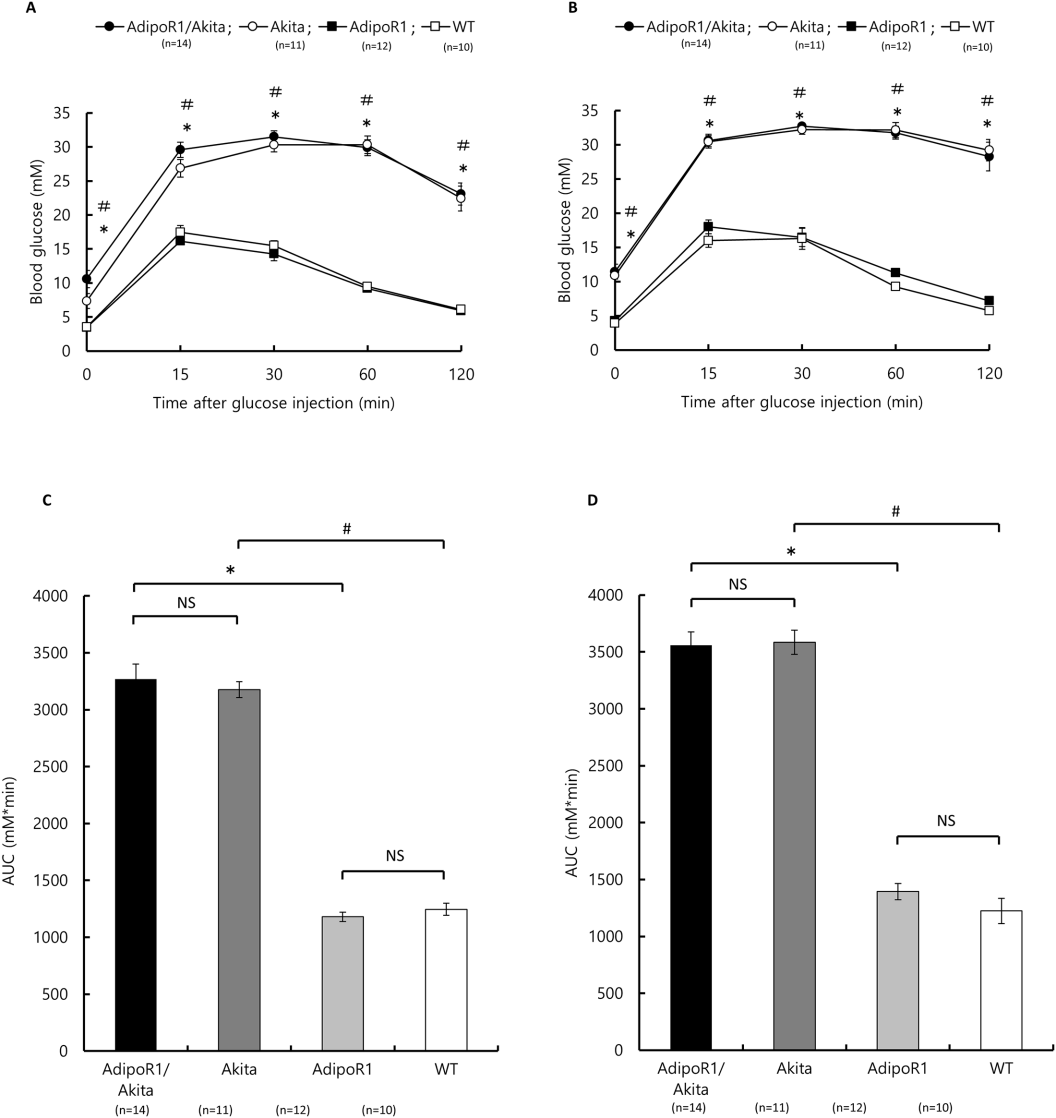
Glucose tolerance of AdipoR1/Akita, AdipoR1, Akita and WT mice. GTT of 6- and 10-weeks-old AdipoR1/Akita (n = 14), Akita (n = 11), AdipoR1 (n = 12) and WT (n = 10) mice. Blood glucose concentrations are shown at indicated times after glucose injection (A, 6-weeks-old; B, 10-weeks-old). Area under the curve (AUC) for GTT was calculated from these results (C, 6-weeks-old; D, 10-weeks-old). Data are shown as mean ± SEM. There were no significant differences between AdipoR1/Akita and Akita, nor between AdiporR1 and WT mice, whereas there was significant differences between AdipoR1/Akita and AdipoR1 mice (* p<0.05), and between Akita and WT mice (# p<0.05) by ANOVA followed by Tukey’s honestly significant difference test. NS, Not significant.

As shown in Fig 4, GTT, using 6- and 10-weeks-old mice, showed that glucose tolerance in AdipoR1/Akita mice (Area under the curve [AUC] 6 weeks, 3270±132 mM*min; 10 weeks, 3558±118 mM*min) was not significantly different from that in Akita mice (AUC 6 weeks, 3177±69 mM*min; 10 weeks, 3584±106 mM*min). There were also no significant differences between AdipoR1 mice (AUC 6 weeks, 1181±41 mM*min, 10 weeks 1394±72 mM*min) and WT mice (AUC 6 weeks, 1246±53 mM*min; 10 weeks 1224±112 mM*min).

### Pancreatic insulin content

**Fig 5:**
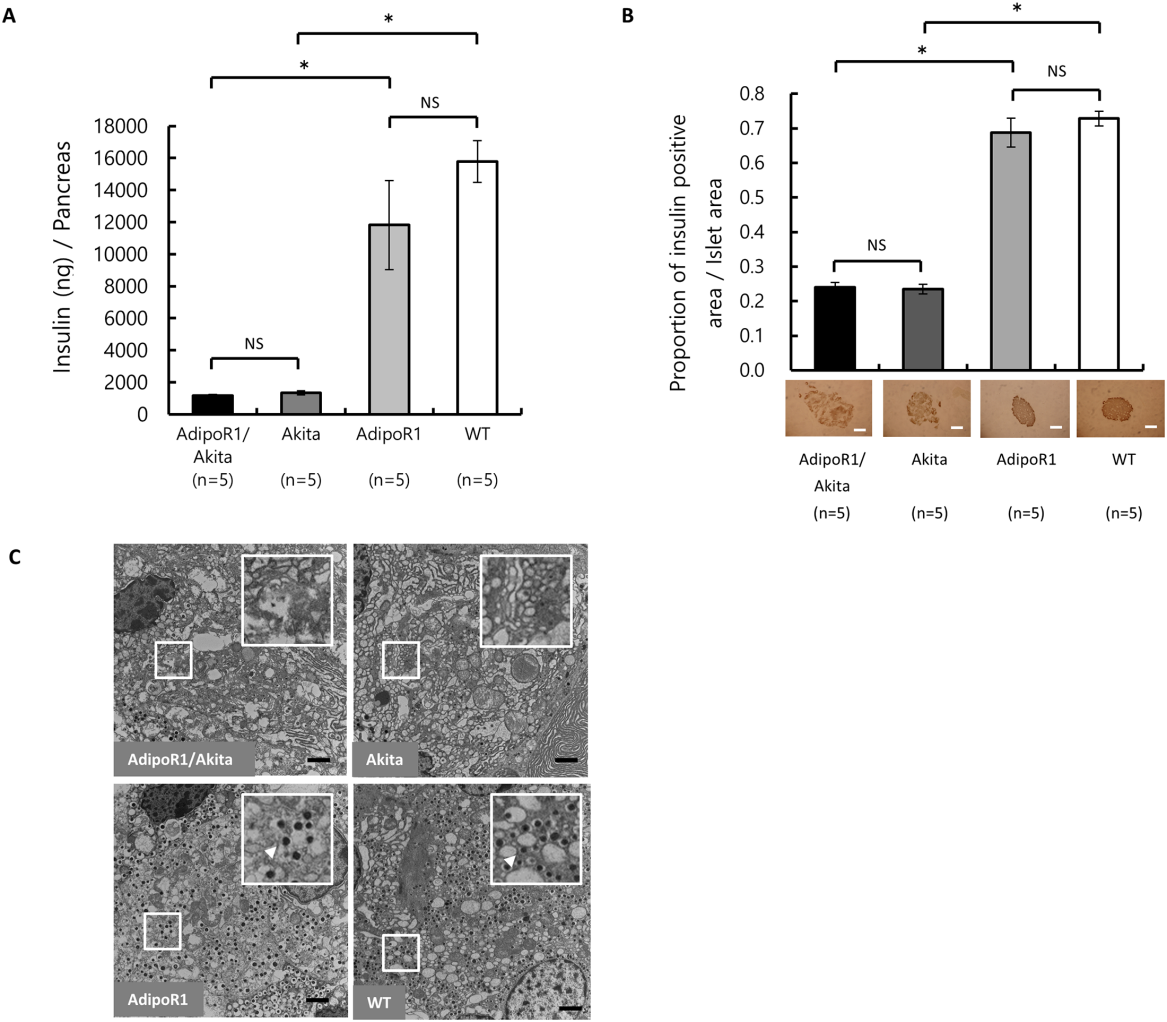
Pancreatic insulin contents and morphological characteristics of AdipoR1/Akita, AdipoR1, Akita and WT mice. Pancreatic tissue samples were obtained from AdipoR1/Akita, AdipoR1, Akita and WT mice at 15–20 weeks of age. (A) Pancreatic insulin contents of AdipoR1/Akita (n = 5), Akita (n = 5), AdipoR1 (n = 5) and WT (n = 5) mice. (B) Insulin positive area per islet area in AdipoR1/Akita (n = 5), Akita (n = 5), AdipoR1 (n = 5) and WT (n = 5) mice (upper panel). Quantification was performed on more than 20 islets from each mouse. Representative images of tissues/islet cells immunostained with anti-insulin antibodies (lower panel). Scale bars indicate 50 μm. (C) Representative images of electron micrographs of islet cells in AdipoR1/Akita, AdipoR1, Akita and WT mice. Similar results were obtained from three independent experiments. Scale bars indicate 2 μm. The small boxed areas have been enlarged and are shown in the corresponding large boxes. Arrows indicate insulin secretory granules. Data are shown as mean ± SEM. There were no significant differences between AdipoR1/Akita and Akita, or between AdipoR1 and WT, whereas there was significant differences between AdipoR1/Akita and AdipoR1 (* p<0.05), and between Akita and WT (# p<0.05) by ANOVA followed by Tukey’s honestly significant difference test. NS, Not significant.

Total pancreatic insulin content, determined using 15-20-weeks-old mice (Fig 5A), demonstrated that pancreatic insulin content did not significantly differ between AdipoR1/Akita mice (1157.86 ± 67.67 ng/pancreas) and Akita mice (1350.16 ± 124.87 ng/pancreas). There were also no significant differences between AdipoR1 mice (11817.22 ± 2782.59 ng/pancreas) and WT mice (15785.20 ± 1309.31 ng/pancreas). However, pancreatic insulin contents of AdipoR1/Akita and Akita mice were significantly lower than those in AdipoR1 mice and WT mice, respectively.

### Insulin immunohistochemistry and electron microscopy of islets

Immunostaining of pancreatic islets with anti-insulin antibody was performed using 15–20 weeks old mice (Fig 5B). AdipoR1/Akita mice (0.241 ± 0.014 insulin positive cells/islet) and Akita mice (0.235 ± 0.014 insulin positive cells/islet) showed a significant decrease in insulin positive cells in pancreatic islets as compared with AdipoR1 mice and WT mice. However, there were no significant differences between AdipoR1/Akita and Akita mice, nor between AdipoR1 mice (0.688 ± 0.042 insulin positive cells/islet) and WT mice (0.728 ± 0.021 insulin positive cells/islet).

Electron microscopy of β-cells in AdipoR1/Akita and Akita mice at 15–20 weeks of age showed similar morphologies, such as a decrease in insulin secretory granules, expansion of the ER, markedly enhanced swelling and disruption of mitochondria, and degeneration of the cytoplasm, whereas AdipoR1 and WT mice at 15–20 weeks of age showed no such morphological abnormalities (Fig 5C).

#### Serum concentration of adiponectin

**Fig 6:**
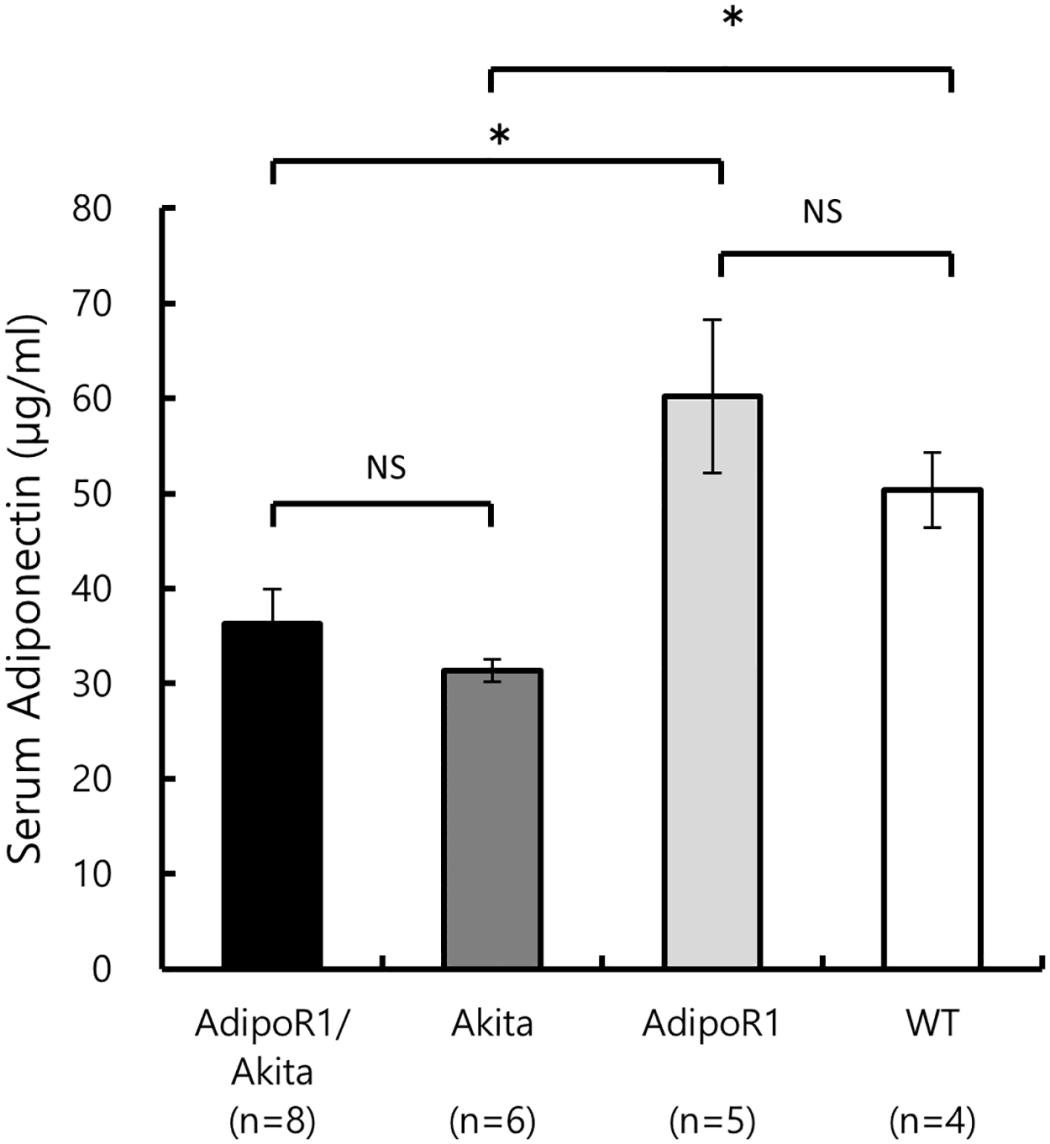
Serum adiponectin levels. Serum adiponectin levels of AdipoR1/Akita (n = 8), AdipoR1 (n = 5), Akita (n = 6) and WT (n = 4) mice at 15–20 weeks of age. Values are expressed as mean ± SEM. *P < 0.05 by ANOVA followed by Tukey’s honestly significant difference test. NS, Not significant.

Serum adiponectin concentrations (Fig 6) showed no significant differences between the AdipoR1/Akita mice (36.3 ± 3.7 μg/ml) and Akita mice (31.4 ± 1.2 μg/ml), nor between the AdipoR1 mice (60.2 ± 8.1 μg/ml) and WT mice (50.4 ± 4.0 μg/ml). However, the adiponectin concentrations in AdipoR1/Akita and Akita mice were significantly lower than those in AdipoR1 mice and WT mice, respectively.

Significant decrease in serum adiponectin levels, however, cannot be causally attributed to the decrease in β-cell mass in AdipoR1/Akita mice because the effects of adiponectin, is already saturated at physiological levels as predicted by Eq 4. Thus observation that the extent of destruction of pancreatic β cells in AdipoR1/Akita mice is similar to that in Akita mice can discard protective effects of adiponectin from apoptosis.

## Discussion

AdipoR1 is known to be expressed in pancreatic β-cells, blood vessels, and in the central nervous system [15], and to be the main receptor for adiponectin action in mice [14]. As adiponectin has been proposed to protect pancreatic β-cells from destruction, we sought to address this function by overexpressing AdipoR1 in β-cells of Akita mice. We first confirmed that expression of the EGFP-tagged AdipoR1 transgene, under control of the rat insulin promoter, which can induce AdipoR1 in concert with insulin demand, was localized to the β-cells of islets, and that the protein was predominantly present in the membrane fraction of transfected cells. We also confirmed that Akt and AMPK were activated when AdipoR1 was overexpressed in MIN6 cells as well as in islets isolated from AdipoR1 transgenic mice, but not in islets isolated from wild type mice in which AdipoR1 is maintained at basal levels. These data collectively illustrate the deterministic role of AdipoR1 as predicted by Eq 4. Despite the correct expression and localization of the AdipoR1 transgene, as well as the adiponectin-induced downstream signaling events, our current study does not support the conjecture that adiponectin protects β-cells from apoptosis in Akita mouse. No significant differences between AdipoR1/Akita and Akita mice were observed in blood glucose level, glucose tolerance, pancreatic insulin content, or insulin-positive areas in pancreatic islets. In agreement with these diabetic phenotypes, electron microscopic observations of pancreatic β-cells also demonstrated that there were no differences in the extent of β-cell mass between AdipoR1/Akita and Akita mice. These data strongly suggest, therefore, that adiponectin does not protect β-cells from apoptosis in Akita mice. Akita mice carry a heterozygous C96Y mutation in the *Ins2* gene and therefore spontaneously develop hyperglycemia, which manifests itself by 6 weeks of age, with reduced pancreatic β-cell mass as a result of ER stress [18, 19].

Akita mouse is a prototype apoptosis model, attributable to ER stress due to a C96Y mutation, which results in failure of A7-B7 disulfide bond formation and leads to the unfolded protein response [20, 21]. There are other causes of ER stress: islet β-cells are sensitive to hyperglycemia (i.e., glucotoxicity) or hyperlipidemia, which can alter the Ca^2+^ concentration and increase the generation of reactive oxygen species due to mitochondrial damage and thereby lead to apoptosis [22]. Yu et al. [23] recently confirmed that the various pathways leading to apoptosis are positively regulated by CHOP-caspase activation signals and negatively regulated by NR4A1-survivin signals. We previously demonstrated that ablation of CHOP, which is activated by ER stress, could partially delay the elevation of blood glucose from 6 weeks until 12 weeks of age in Akita mice [24]. However, in our current study, we could not detect any sign of amelioration of hyperglycemia in the AdipoR1/Akita mice, indicating that β-cell-specific overexpression of AdipoR1 failed to protect pancreatic β-cells from apoptosis. Such failure may suggest that either adiponectin does not have anti-apoptotic protective effects on β-cells *in vivo*, or that the apoptotic signals in Akita mice are so strong that they overwhelm the protective effects of adiponectin. Data are limited at present and future studies are required to address these possibilities.

In this study, serum adiponectin was measured to verify the extremely high adiponectin blood concentration values reported in the literature. There were no significant differences in serum adiponectin levels between AdipoR1/Akita mice and Akita mice, but these were significantly lower than levels in AdipoR1 and WT mice even though the levels in both groups were more than 30 μg/ml. These extremely high serum adiponectin concentrations, compared to the reported Kd value (0.06 μg/ml) [14], further confirm the validity of our hypothesis that established Eq 2 (refer to Introduction section).

Although adiponectin is a protein that is expressed and secreted specifically in adipocytes, it has been reported that there is a decrease in adiponectin concentration in blood due to obesity [25]. However, lower adiponectin levels were not observed in some obese mouse models that displayed abnormal lipid metabolism [26]. In this study, AdipoR1/Akita mice and Akita mice, which had low body weights, also had lower adiponectin concentrations compared with the AdipoR1 mice and WT mice, suggesting that blood adiponectin levels in Akita mice may be influenced by factors other than fat mass. However, such decreased levels of adiponectin do not appear to elicit any biological effects through the AdipoR1 signaling pathway.

In summary, we have confirmed in this study that adiponectin can activate downstream Akt and AMPK signaling pathways only when AdipoR1 is overexpressed in islet β-cells, suggesting that adiponectin may improve insulin resistance when AdipoR1 is induced in muscle or adipose tissues. However, pancreatic β-cell-specific overexpression of AdipoR1 does not have protective effects on ER stress-induced β-cell destruction in Akita mice. Thus, the use of the anti-apoptotic effects of adiponectin on pancreatic β-cells may not be justified for pharmacological applications.
